# SUBJECTIVE AGING: SOMETHING UNIQUE OR JUST ANOTHER EXPRESSION OF GENERAL SELF-BELIEFS?

**DOI:** 10.1093/geroni/igz038.2895

**Published:** 2019-11-08

**Authors:** Verena Klusmann, Svenja M Spuling, Catherine E Bowen, Anna E Kornadt, Eva-Marie Kessler

**Affiliations:** 1 University of Konstanz, Konstanz, Germany; 2 German Center of Gerontology, Berlin, Berlin, Germany; 3 Independent Researcher Scientific Network Images of Aging, Vienna, Wien, Austria; 4 Bielefeld University, 33615, Nordrhein-Westfalen, Germany; 5 Medical School Berlin, 12247, Berlin, Germany

## Abstract

Using data from the German Ageing Survey (adults aged 40‒85), this study tested the convergent and discriminant validity of subjective aging measures by comparing three different measures of subjective aging with one another and relating them to established measures of general self-beliefs (optimism, self-efficacy, subjective health) and subjective well-being (depression, affect). Correlations between subjective aging measures ranged from ‒.61 (amongst general self-perceptions of aging measures) to ‒.09, with subjective age being least related to the other measures. The highest overlap was observed between optimism and global self-perceptions of aging (.69) and it was for these global self-perceptions that the highest amount of variance could be explained by correlates in a regression analysis (R-square=.55). In contrast, only 10% of variance could be explained for subjective age. Our results underline the merit of taking the multidimensional nature of subjective aging into account since global measures appear less distinct from general personality traits.

